# Structural determinants of suicide during the global financial crisis in Spain: Integrating explanations to understand a complex public health problem

**DOI:** 10.1371/journal.pone.0247759

**Published:** 2021-03-01

**Authors:** Javier Alvarez-Galvez, Victor Suarez-Lledo, Luis Salvador-Carulla, Jose Almenara-Barrios

**Affiliations:** 1 Department of Biomedicine, Biotechnology and Public Health, University of Cadiz, Cadiz, Spain; 2 Centre for Mental Health Research, Research School of Population Health, Australian National University, Canberra, Australia; 3 Menzies Centre for Health Policy and Economics, School of Public Health, Faculty of Medicine and Health, University of Sydney, Camperdown, Australia; University of California-Irvine, UNITED STATES

## Abstract

**Introduction:**

Suicide is a complex public health problem in contemporary societies. Macroeconomic downturns derived from the economic crisis have been found to be associated with growing suicide mortality in the United States and in Europe. The present work is aimed to assess the association between the recent economic downturns and suicide patterns using interrupted time series analysis and, particularly, adjusting this relationship by indicators of social cohesion and community values that might provide additional insights on the complex explanation of suicidal trends.

**Methods:**

We combined suicide, social and economic data extracted from the *National Statistics Institute (INE)*, the *Eurostat database*, and the *World Values Survey* to assess the association between the socio-economic factors and trends in suicide rates. To study the association between the financial crisis and changes in suicide rates in Spain, we used interrupted time series analysis (ITSA).

**Results:**

Our findings confirm that suicides increased after the 2011 recession, but remained moderately constant after the 2008 economic downturn. Suicides particularly increased after the 2011 recession in the 10–14, and 45–64 years old intervals between males and females, and apparently in older groups. However, during the 2008–2011 time period suicide rates decreased during working years (specifically among 40–44, 45–49, and 55–59 years old groups). Our results highlight the importance of social protection against unemployment and, to a lesser extent, social protection in disability and family, in reducing suicides, as well as the economic prosperity of the country.

**Conclusion:**

This result corroborates that the economic crisis has possibly impacted the growing suicide rates of the most vulnerable groups, but exclusively during the period characterised by economic cuts after the 2011 recession. This study highlights the need to implement tailored policies that protect these collectives against suicide.

## Background

Suicide is a complex public health problem in contemporary societies [1, 2]. Due to the 2008 financial crisis, the difficulties for addressing this challenging research topic has even increasing [3, 4]. Macroeconomic downturns derived from the economic crisis have been found to be associated with growing suicide mortality in the United States and in Europe [3, 5–7]. In Europe, southern countries such as Greece, Italy, Portugal or Spain have been deeply impacted by unemployment and austerity policies [8, 9], and recent studies have revealed that variations in these macroeconomic indicators are positively correlated with suicides [3, 10–13]. However, as some studies have confirmed [4], the supposed relationship between economic recession and increasing suicide rates is under debate in specialised literature.

In particular, three questions of controversy have been found: (1) which are the periods of association and the possible starting points? [14, 15]; (2) which are the macroeconomic determinants that explain variations in suicide rates? [16]; and (3) which are the most suitable methods (i.e. analytical techniques, data sources, etc.) to answers the previous questions? [17]. These questions have been addressed differently by several studies and for different countries with more or less success.

In relation to the first point of disagreement, some studies have identified a statistically significant association between the macroeconomic problems and suicides rates from the very beginning of the crisis in the year 2008 [10, 15], while other studies describe increasing suicide mortality as a delayed effect that emerged during the subsequent years [18]. Some studies have described a double-dip suicide rate association with the corresponding double-period recession in Spain [i.e. one in the year 2008 and the other on the years 2011], although other research works only found a significant relationship with the second recession [18, 19]. In a same vein, current findings in Hungary suggest that unemployment and austerity policies might have had a delayed impact on suicidality with an effect appearing after 3–5 years [4, 20]. The second point of controversy is related to the macroeconomic determinants that might explain increasing suicide rates. Some have generally focused on unemployment as one of the most relevant causes of suicides during the period of economic crisis [11, 12, 21], but this social determinant has also led to inconsistent findings [16]. Lastly, the last point of disagreement is associated to the methodological difficulties in the comparison and analysis of suicide data. Studies have used different methods to study this complex social phenomenon, but also different types suicide data (crude suicide rates, age-standardised rates, suicide ideation, etc.) at different level of aggregation depending on data availability [17].

In addition, while many studies have been looking for economic explanation of increasing suicide rates during and after the global financial crisis, less attention has been paid to understand the relationship with other factors that compose the essential matter of societies’ structure. Health sciences have mainly focused on the macroeconomic determinants of an economic nature, however, these researchers have put less interest in understanding other social factors that may explain suicide as a multidimensional phenomenon. In fact, recent evidence and, in particular, the emergence of new right wings parties and nationalist movements suggests that negative personal experiences suffered during the economic downturn have undermined generalized trust and social relations between the different socioeconomic groups [22]. Consequently, the financial crisis has not only eroded the economic system and the welfare protection system, but also has impacted on fundamental societal constituents such as interpersonal trust, social capital or changes in post-materialist values that might be directly or indirectly involved in the explanation of increasing suicide trends [23]. For instance, recent studies have showed that communities with higher levels of social capital (i.e. social cohesion) had significantly lower rates of suicide [24], while post-materialist values, understood as value orientation that emphasizes self-expression and quality of life over economic and physical security, might become a society more permissive with suicidal behavior [25, 26].

Using Spain as a case study that can be representative of these theoretical and methodological gaps in literature, the present work is aimed to assess the association between the recent economic downturns and age-standardised suicide rates using interrupted time series analysis and, particularly, adjusting this relationship by indicators of social cohesion and community values that might provide additional insights on the complex explanation of suicidal trends. According to these main objectives, our study is grounded on two central hypotheses: (H1) The association between the economic downturn and suicide rates during the second recession period is limited to specific sex and age groups; and (H2) societal indicators such as interpersonal trust, social capital or post-materialist values should moderate the association between economic downturns and suicides.

## Material and methods

### Data and variables

In this study, we are using data from three main data sources. First, for our dependent variable, we collated suicide rates per 100,000 inhabitants as provided by the *National Statistics Institute (in Spanish*, *Instituto Nacional de Estadística–INE)*. We transformed crude rates in age-standardised suicide rates to make comparisons and to account for the differences in the age structure of the populations being studied according their sex and age group. Second, three macro-economic indicators extracted from the *Eurostat database* were included in the analysis as independent variables to assess the association between the financial crisis factors and trends in suicide rates. We used three predictors of social inequalities in mental illness and suicide: (1) unemployment rate; (2) gross domestic product growth (GDP growth); and (3) social expenditure (in percentage of GDP), included here as a proxy measure of the size of the welfare state. Finally, three additional indicators related to social relationships and societal values were collated from the *World Values Survey*: (4) interpersonal trust; (5) social capital and (6) post-materialist values. Descriptive statistics for variables used in the ITSA models are presented in Table 1.

**Table 1:** Descriptive statistics.

**Age-Std. suicide rates**	**N**	**Mean**	**Std. Dev.**	**Min**	**Max**
A-S suicide rate 10–14 y.o.	76	0.014	0.013	0.000	0.052
A-S suicide rate 15–19 y.o.	76	0.091	0.065	0.013	0.261
A-S suicide rate 20–24 y.o.	76	0.195	0.148	0.019	0.523
A-S suicide rate 25–29 y.o.	76	0.231	0.170	0.040	0.599
A-S suicide rate 30–34 y.o.	76	0.246	0.168	0.042	0.668
A-S suicide rate 35–39 y.o.	76	0.263	0.167	0.048	0.567
A-S suicide rate 40–44 y.o.	76	0.271	0.177	0.058	0.631
A-S suicide rate 45–49 y.o.	76	0.280	0.173	0.072	0.725
A-S suicide rate 50–54 y.o.	76	0.298	0.166	0.084	0.740
A-S suicide rate 55–59 y.o.	76	0.289	0.147	0.105	0.555
A-S suicide rate 60–64 y.o.	76	0.278	0.130	0.103	0.502
A-S suicide rate 65–69 y.o.	76	0.273	0.126	0.077	0.508
A-S suicide rate 70–74 y.o.	76	0.281	0.130	0.099	0.561
A-S suicide rate 75–79 y.o.	76	0.276	0.140	0.090	0.519
A-S suicide rate 80–84 y.o.	76	0.222	0.127	0.035	0.442
A-S suicide rate 85+ y.o.	76	0.180	0.114	0.029	0.453
A-S suicide rate (Total)	76	3.687	1.952	1.107	6.347
**Socio-economic vars.**	**N**	**Mean**	**Std. Dev.**	**Min**	**Max**
Social expenditure (%)	76	20.353	2.958	15.000	25.800
Unemployment rate (%)	76	17.673	4.889	8.260	25.770
GDP growth	76	2.345	2.240	-3.600	5.500
Post-Mat. Values	76	2.485	0.219	2.212	2.800
Trust index	76	28.083	4.237	18.920	32.750
Social Capital	76	0.664	0.018	0.633	0.683

### Statistical analysis

To study the association between the financial crisis and changes in suicide rates in Spain, we used interrupted time series analysis (ITSA). This technique was aimed to examine, on the one hand, the effect of the economic recession over suicide trends during the period ranged from 1980 to 2017, and in the other hand, to analyse the variations in suicide rates linked to macro-economic fluctuations (i.e. in unemployment, GDP growth, and social expenditure) and social changes measured by interpersonal trust, social capital and post-materialist values. Although the recent studies have been mainly focused in the recession period of the 2008 Financial crisis (i.e., a first recession in the beginning of the crisis during the years 2008–2010, and the second one during 2011–2013, where the austerity policies of the Troika were implemented), here we also incorporate the recession of 1992–1993 to increase our understanding of the relationship between economic crisis and suicides.

In contrast with other analytical approaches, the ITSA permitted to work with data series that present comparability problems due to missing information or methodological problems in data collection [i.e. data series gaps], but also explore how contextual events (e.g., the application of certain interventions, programmes, policies, etc.) might explain variation in data trends that could affect in the interpretation of the findings. In this case, since we have two groups available for comparison, we will use the ITSA model for multi-group analysis [27–30]. In our work, the multiple-group ITSA is especially valuable because we are hypothesizing that the 2008 economic downturn might have differently impacted between males and females, and so in diverse age groups. The model to be implemented is described below:

$$Y_{t}=\beta_{0}+\beta_{1}T_{t}+\beta_{2}X_{t}+\beta_{3}X_{t}T_{t}+\beta_{4}Z+\beta_{5}ZT_{t}+\beta_{6}ZX_{t}+\beta_{7}ZX_{t}T_{t}+\epsilon_{t}$$

Alike to other regression models, *Y*_*t*_ is the dependent variable measured at each similarly spaced time *t*, while the time elapsed from the beginning of the study is represented by *T*_*t*_, the intervention [or contextual change] is defined by a binary indicator *X*_*t*_, Z is a dummy variable to designate the cohort assignment (i.e. treatment or control group), and *X*_*t*_*T*_*t*_, *ZT*_*t*_, *ZX*_*t*_, and *ZX*_*t*_*T*_*t*_ are the interaction terms among previously mentioned variables. The coefficients *β*_*0*_ to *β*_*3*_ represent the control group intercept and slopes, and *β*_*4*_ to *β*_*7*_, represent values of the treatment group [30].

## Results

To study the trajectory of age-standardised suicide rates in Spain, the multi-group ITSA model was conducted with the objective of analysing the changes in suicide trends in Spain linked to different contextual intervals, defined by three economic recession experimented during since 1980 to 2017, and precisely in the years 1992, 2008, and 2011 [18]: (1) the evolution of the initial data series of age-standardised suicide rates from 1980 to 1991 was analysed; (2) the period from 1992 until 2007; (3) the period from 2008 to 2010; and finally (4) the period from 2011 to 2017.

### Suicide trends in Spain

**Fig 1:**
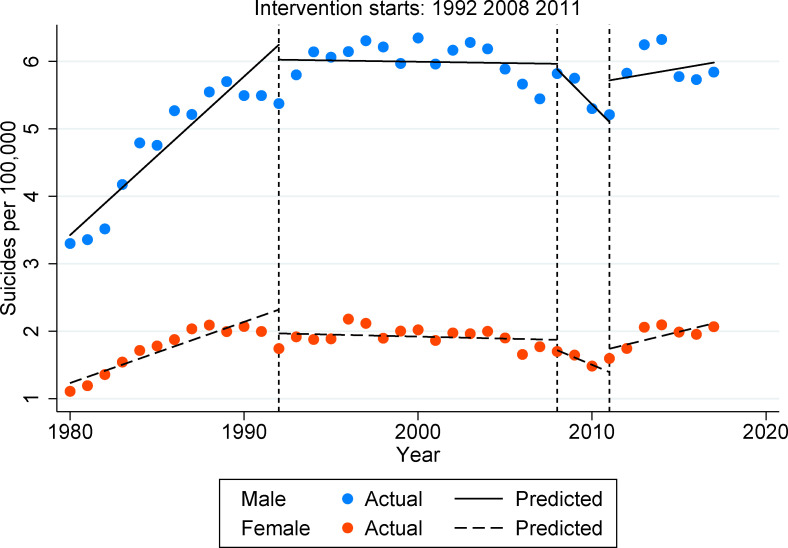
Age-standardized suicide rates in Spain (all age groups).

Fig 1 shows the four periods that characterise suicide trends in Spain during the period 1980–2017, where recession periods (1992, 2008, 2011) are depicted by horizontal dashed lines. First, we can observe that suicided trends significantly grow during the 80s decade. Second, the 1992 recession seems to initially increase suicide trends in males, although rates indistinctly vary during the years from 1992 to 2007, with higher and lower peaks at different points of the period. Among females, suicide trends remain relatively constant during these years. The recession did not show a statistically significant variation either in male and female groups during this period. Third, during the period in which the 2008 economic downturn occurred, the analysis shows a statistically significant reduction in age-standardised suicide rates both for men and woman. Finally, as hypothesised the suicide trends grow during the last period (2011–2017), which coincides with the 2011 economic recession, and particularly with a radical change in social and public policies of the new government that were accords with the full establishment of the Troika policies for economic budget cuts. However, this growing suicide trend is only statistically significant among females (see S1 Table).

### Comparison across age groups

**Fig 2:**
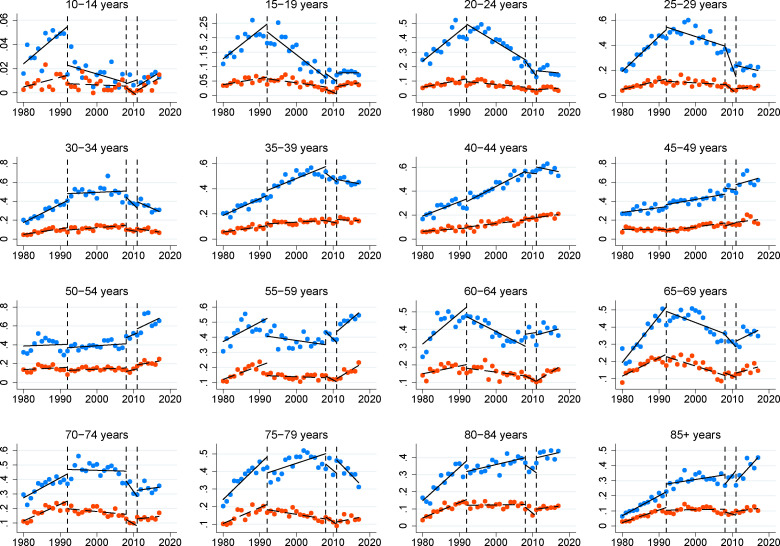
Age-standardized suicide rates in Spain per age groups (linear fit).

Once the general suicide trend has been described for the whole country, it is interesting to observe age related variations in this national pattern (Fig 2). Among the younger group (10–14 years old), suicides have significantly reduced between males and females during from the 1992 economic downturn, and increased after the 2011 recession in both groups (p < 0.01). The same trend can be observed in females between 15 and 29 years old, although this pattern is only statistically significant among the 15–19 (p < 0.05) and 25–29 years old groups (p < 0.10), a particularly in females. Between 30 and 39 years old, suicides intensely grow after the 1992 recession, but the 2008 recession inverted this trend (p < 0.05). From the 40–44 years old group until the 85+ group, suicides seem to reduce since the first 1992 recession, a trend that follows after the 2008 downturn. However, this decreasing suicide pattern vary from the year 2011, the point where suicides start to grow among all groups and specifically in females, the group where this trend present more statistical significance (p < 0.05). Along this common pathway, we can only observe a different pattern in the 75–79 years old group (Table 2).

**Table 2:** Trends in suicides per age-standardized groups.

		Period: 1992–2008		Period: 2008–2011		Period: 2011–2017	
		Coef.	P>t	Coef.	P>t	Coef.	P>t
10–14 yo	Male	-0.001	0.0365	0.001	0.686	0.002	0.002
	Female	<-0.001	0.619	-0.002	0.000	0.002	0.001
15–19 yo	Male	-0.010	0.000	-0.009	0.296	<0.000	0.909
	Female	-0.002	0.141	-0.006	0.000	0.002	0.050
20–24 yo	Male	-0.015	0.000	-0.035	0.000	-0.003	0.667
	Female	-0.002	0.056	-0.002	0.599	0.002	0.581
25–29 yo	Male	-0.001	0.022	-0.081	0.000	-0.007	0.053
	Female	-0.001	0.203	-0.024	0.000	0.003	0.088
30–34 yo	Male	0.002	0.694	-0.039	0.000	-0.017	0.000
	Female	0.003	0.001	-0.007	0.002	-0.006	0.000
35–39 yo	Male	0.012	0.001	-0.023	0.020	-0.006	0.097
	Female	0.002	0.001	-0.006	0.382	-0.001	0.546
40–44 yo	Male	0.016	0.000	-0.004	0.740	-0.006	0.242
	Female	0.004	0.023	0.003	0.278	0.004	0.155
45–49 yo	Male	0.007	0.000	-0.004	0.764	0.011	0.400
	Female	0.005	0.000	0.012	0.037	0.005	0.562
50–54 yo	Male	0.003	0.291	0.016	0.128	0.019	0.163
	Female	0.002	0.227	0.009	0.293	0.007	0.066
55–59 yo	Male	-0.004	0.101	-0.024	0.000	0.021	0.003
	Female	-0.001	0.258	-0.013	0.000	0.015	0.000
60–64 yo	Male	-0.010	0.000	0.003	0.833	0.006	0.498
	Female	-0.003	0.027	-0.011	0.000	0.013	0.000
65–69 yo	Male	-0.008	0.035	-0.024	0.000	0.011	0.092
	Female	-0.007	0.002	-0.008	0.026	0.006	0.021
70–74 yo	Male	-0.001	0.871	-0.036	0.007	0.004	0.444
	Female	-0.002	0.020	-0.015	0.000	0.003	0.333
75–79 yo	Male	0.007	0.030	-0.018	0.403	-0.022	0.000
	Female	-0.003	0.002	-0.014	0.000	0.004	0.245
80–84 yo	Male	0.005	0.000	-0.015	0.132	0.005	0.256
	Female	<0.001	0.657	-0.018	0.070	0.003	0.001
85+ yo	Male	0.004	0.069	0.031	0.000	0.027	0.000
	Female	<0.001	0.794	-0.007	0.088	0.003	0.081

### Social and economic determinants of suicides

Once we have reached this point, one inevitable research question is: which are the social and economic factors behind increasing suicides after the 2011 recession period? Many studies have generally focused on unemployment as one of the most relevant causes of suicides after the 2008 economic downturn, but this social determinant has also led to inconsistent findings [31]. Trying to find alternative explanations to increasing suicides after the 2011 recession, here we explore how changes in social structures might have covaried with suicidal trends during the economic downturns.

Table 3 show the adjusted models using six possible macrostructural factors (i.e. social expenditure, unemployment, GDP growth, Post-materialist values, trust, and social capital) to explore the social and economic determinants of suicide trends. Among the economic determinants we can observe that unemployment presents a positive relationship with suicides, i.e. the higher the country-level unemployment the higher the suicide rates. In particular, this association is statistically significant between the age groups between 40 and 64 years old (i.e., during working years), but this relationship can also be found between older groups (e.g., 70–74 years old, and 80–84 years old). GPD growth seems to be positively associated with suicides, but this association is only statistically significant in the 15–19 and 60–64 years old groups. Social expenditure did not present a regular association pattern.

**Table 3:** The relationship between suicide patterns and structural determinants.

**Structural determinants**	**10–14**	**15–19**	**20–24**	**25–29**	**30–34**	**35–39**	**40–44**	**45–49**	**50–54**	**55–59**	**60–64**	**65–69**	**70–74**	**75–79**	**80–84**	**85+**	**Total**
Social expenditure	0.000	0.001	0.002	-0.001	0.002	0.000	0.001	**0.004**	0.000	0.000	0.000	-0.001	-0.002	**-0.002**	0.000	0.000	0.003
Unemployment rate	0.000	0.000	0.000	0.000	0.000	0.000	**0.001**	**0.001**	**0.002**	**0.001**	**0.002**	0.000	**0.001**	0.000	**0.001**	0.000	**0.011**
GDP growth	0.000	**0.000**	0.000	0.000	0.000	0.000	0.000	0.000	0.000	0.000	**0.000**	0.000	0.000	0.000	0.000	0.000	0.002
Post-Mat. Values	0.000	-0.001	-0.001	-0.001	0.000	-0.001	0.001	0.000	**0.002**	**0.002**	**0.001**	**-0.002**	**-0.002**	0.000	0.000	**-0.001**	-0.004
Trust index	0.000	0.000	0.000	0.000	0.000	0.000	**0.001**	**0.001**	**0.001**	0.001	**-0.001**	0.000	0.000	0.000	0.000	0.000	0.003
Social Capital	0.000	**0.001**	0.001	**0.003**	0.001	0.001	**-0.001**	**-0.001**	**-0.002**	**-0.002**	0.000	**0.002**	**0.002**	**0.002**	-0.001	0.001	0.006
Constant	0.003	-0.030	0.007	-0.066	-0.025	0.037	-0.018	0.099	-0.056	0.006	-0.037	0.064	0.057	-0.066	0.035	-0.001	0.259
**Suicide patterns**																	
***Period*: *1992–2008***	**10–14**	**15–19**	**20–24**	**25–29**	**30–34**	**35–39**	**40–44**	**45–49**	**50–54**	**55–59**	**60–64**	**65–69**	**70–74**	**75–79**	**80–84**	**85+**	**Total**
Male	-0.001	**-0.009**	**-0.013**	**-0.010**	0.005	**0.014**	**0.023**	**0.017**	**0.021**	**0.010**	0.002	**-0.009**	0.002	**0.011**	**0.010**	**0.004**	**0.066**
Female	0.000	-0.001	0.000	-0.002	**0.006**	**0.004**	**0.011**	**0.015**	**0.020**	**0.013**	**0.010**	**-0.007**	0.000	0.000	**0.005**	0.001	**0.065**
***Period*: *2008–2011***																	
Male	-0.006	0.0033	-0.066	-0.006	-0.058	-0.031	**-0.062**	**-0.141**	-0.071	**-0.086**	-0.006	-0.001	0.003	**0.065**	-0.044	0.022	**-0.493**
Female	-0.009	0.005	-0.033	0.045	-0.037	-0.016	**-0.055**	**-0.125**	-0.080	**-0.071**	-0.016	0.014	0.025	**0.063**	-0.041	-0.007	-0.334
***Period*: *2011–2017***																	
Male	**0.003**	-0.001	0.002	-0.009	**-0.014**	-0.001	-0.003	**0.031**	**0.032**	**0.036**	**0.014**	0.006	0.003	**-0.022**	0.006	**0.025**	**0.123**
Female	**0.003**	0.0006	0.007	0.000	-0.001	0.003	0.008	**0.025**	**0.020**	**0.030**	**0.021**	0.002	0.002	**0.002**	**0.006**	0.003	**0.133**

Note: Coefficients in bold are statistically significant at the level of p <0.05.

Country-level indicators related to social cohesion and norms also were found to be statistically associated with suicide patterns. The post-materialist values indicator was positively associated with suicides among the groups between the 40–74 years interval (either at the level of p < 0.05 or p < 0.10), but also in the 85+ group (p < 0.05). Interpersonal trust was also positively related to suicide rates, but just along the 40–59 interval (p < 0.05). Finally, social capital as an essential indicator of social cohesion presented two distinctive patterns. This indicator was found to be negatively associated with suicides among the 40–59 years old interval, but positively associated among younger and older groups (e.g. 15–19 and 25–29 years old groups, and all along the 65–79 years interval).

Below the covariates we can observe that the adjusted models confirm the general suicide pattern in Spain, i.e. suicides increased after the 2011 recession (as did after the 1992 recession), but remained moderately constant in the beginning of the 2008 economic downturn. When we explore age group differences, the general pattern presents some peculiarities that are fundamental to mention. Suicides particularly increased after the 2011 recession in the 10–14 and 45–64 years old intervals between males and females, and also in older groups (though the effect is not that clear). However, during the 2008–2011 years interval suicide rates decreased during working years, specifically among 40–44, 45–49, and 55–59 years old groups.

**Fig 3:**
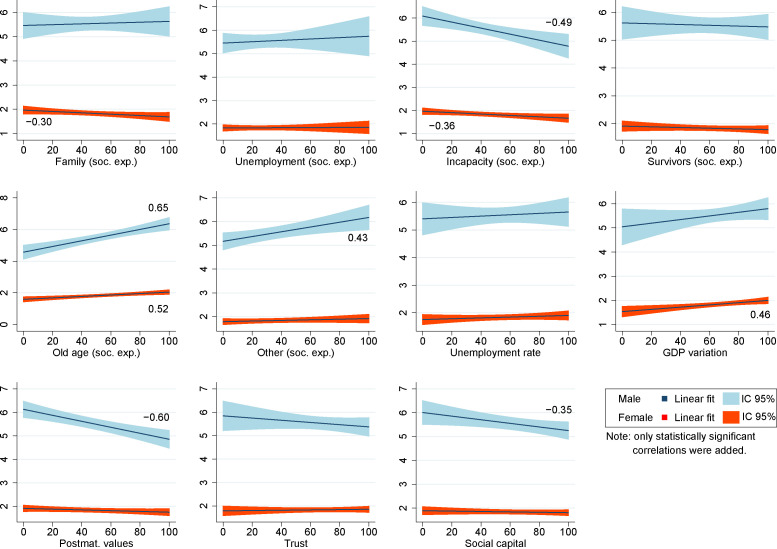
Bivariate association between indicators and age-standardized suicide rates (linear fit).

Finally, with the intention of investigating in greater depth the relationship between social expenditure and the age-adjusted suicide rates, we proceeded to include social expenditure disaggregated according to the different branches of spending (families, unemployment, survivors, old age, incapacity, and others). Before including these indicators, we proceeded to analyze the bivariate relationships of these indicators with age-standardized suicide rates with the idea of evaluating the appropriateness of disaggregating social spending (Fig 3). This correlation analysis allowed us to observe the existence of some negative relationships which indicated that branches of social spending–such as those aimed to families or incapacity–could have a reducing effect on suicide rates, while social spending in old age showed a positive relationship. In general, the rest of the indicators presented relationships that were in line with the results in Table 3, although there were also some differences that were clearly related to the analysis in interrupted series.

Once the possibility of including the new social expenditure indicators was studied, all the previous models were implemented again (Table 4). Although the inclusion of these six social spending indicators would lead to certain variations in the results, we can say that, in general terms, the initial logic was maintained. On the one hand, unemployment still showed a positive relationship with the suicide rate and also social capital showed a positive association with suicides among adolescents and young adults (15–39 years old), and the effect of GDP growth was now better defined in the overall model (i.e., as might be expected, higher economic growth reduced suicides). On the other hand, the new social spending indicators also revealed some relevant associations. Probably the most striking association would be between social spending on unemployment and suicide rates. As observed, social expenditure on unemployment showed a reducing effect on age-standardized suicide rates that was present both in the global model and in the models by age groups, i.e. a result that complemented the logic of the positive effect of unemployment on the increase of suicide rates.

**Table 4:** The relationship between suicide patterns and structural determinants with disaggregated social expenditure.

**Structural determinants**	**10–14**	**15–19**	**20–24**	**25–29**	**30–34**	**35–39**	**40–44**	**45–49**	**50–54**	**55–59**	**60–64**	**65–69**	**70–74**	**75–79**	**80–84**	**85+**	**Total**
Family (Soc. exp.).	0.000	0.001	0.000	0.001	0.000	0.001	0.000	0.001	0.000	0.000	0.001	**-0.001**	-0.001	0.001	0.001	0.001	0.002
Unempl. (Soc. exp.).	0.000	**-0.001**	**-0.001**	-0.001	0.000	**-0.001**	-0.001	-0.001	**-0.001**	0.000	0.000	-0.001	0.000	-0.001	0.000	-0.001	**-0.009**
Incapacity (Soc. Exp)	0.000	0.000	0.000	0.000	-0.001	**-0.001**	0.000	0.000	0.000	-0.001	0.000	0.000	0.000	0.000	0.000	0.000	**-0.004**
Survivors (Soc. Exp.)	0.000	0.000	0.000	0.000	-0.001	0.000	0.000	0.001	0.000	0.000	0.000	0.000	0.000	0.001	0.000	**0.001**	0.004
Old age (Soc. Exp.)	0.000	-0.001	-0.001	0.000	-0.003	**-0.002**	0.000	0.002	-0.002	-0.001	0.000	0.000	-0.001	0.002	-0.002	**0.002**	-0.003
Other (Soc. Exp.)	0.000	0.002	0.002	-0.003	0.001	0.000	0.001	0.002	0.002	0.001	0.000	**0.005**	0.000	-0.003	-0.001	0.000	0.010
Unemployment rate	0.000	**0.001**	0.001	0.001	0.002	**0.001**	0.001	0.000	**0.003**	**0.002**	**0.002**	-0.001	**0.001**	0.000	**0.002**	0.000	**0.012**
GDP growth	0.000	0.000	-0.001	0.000	-0.001	**-0.001**	0.000	-0.001	-0.001	-0.001	0.000	-0.001	0.000	0.000	0.000	0.000	**-0.005**
Post-Mat. Values	0.000	-0.001	-0.002	0.000	**-0.003**	**-0.002**	0.001	0.000	0.001	0.001	**0.002**	-0.001	**-0.002**	0.001	0.000	0.000	-0.005
Trust index	0.000	0.000	0.000	-0.001	0.000	0.000	0.000	**0.001**	**0.001**	0.000	**-0.001**	**0.001**	0.000	0.000	0.000	0.000	0.001
Social Capital	0.000	**0.002**	**0.002**	0.001	**0.002**	**0.001**	-0.001	0.000	-0.001	-0.001	0.000	**0.002**	0.002	0.000	-0.001	0.000	0.008
Constant	0.002	-0.029	0.038	-0.037	0.130	0.082	-0.002	-0.091	0.017	0.097	-0.071	0.145	0.184	-0.068	0.121	-0.056	0.491
**Suicide patterns**																	
***Period*: *1992–2008***	**10–14**	**15–19**	**20–24**	**25–29**	**30–34**	**35–39**	**40–44**	**45–49**	**50–54**	**55–59**	**60–64**	**65–69**	**70–74**	**75–79**	**80–84**	**85+**	**Total**
Male	-0.002	**-0.019**	**-0.024**	-0.012	-0.006	0.003	0.013	0.004	0.005	0.001	-0.003	**-0.023**	0.003	0.011	0.007	-0.003	-0.039
Female	-0.002	**-0.011**	-0.011	-0.004	-0.005	-0.007	0.001	0.002	0.004	0.004	0.004	**-0.021**	0.001	0.000	0.001	-0.006	-0.040
***Period*: *2008–2011***																	
Male	0.001	**0.038**	-0.015	-0.010	-0.024	-0.023	**-0.043**	**-0.052**	**-0.055**	**-0.088**	-0.003	-0.017	-0.027	0.019	**-0.047**	0.026	**-0.392**
Female	-0.002	**0.039**	0.019	0.039	-0.002	-0.008	-0.036	-0.035	**-0.065**	**-0.071**	-0.012	-0.001	-0.004	0.017	-0.045	0.000	**-0.232**
***Period*: *2011–2017***																	
Male	0.001	-0.001	-0.007	-0.002	0.008	0.010	-0.010	-0.005	**0.038**	**0.048**	0.009	-0.012	**0.021**	**-0.023**	**0.027**	0.008	**0.090**
Female	0.001	0.000	-0.003	0.007	0.021	**0.014**	0.000	-0.011	0.026	**0.042**	**0.017**	-0.016	0.020	0.002	**0.027**	-0.014	**0.100**

Note: Coefficients in bold are statistically significant at the level of p <0.05.

In addition, other interesting associations were found, such as the negative relationship between social spending on disability and suicides. However, this association was only evident in the overall model for the country as a whole (i.e., total model in the last column). The relationship of post-materialistic values, interpersonal trust and social capital was maintained to some extent, although the directions of the relationships were not constant across all age groups.

**Fig 4:**
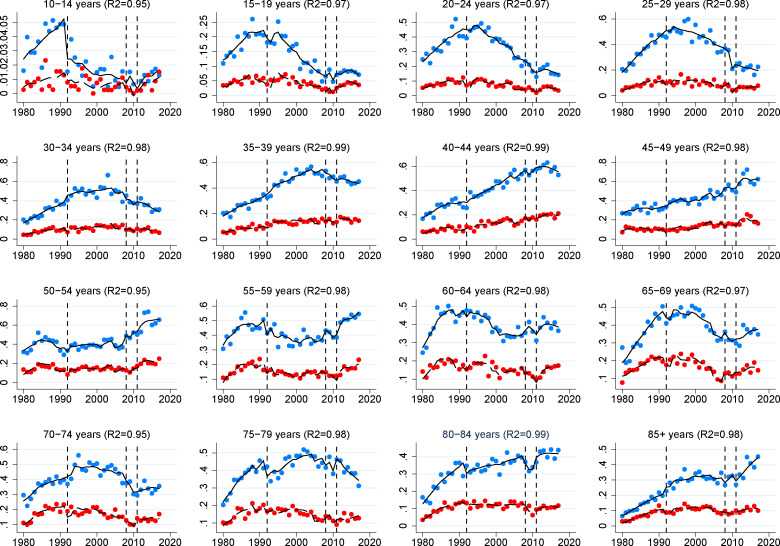
Global adjustment of the ITSA models by age groups.

The indicators introduced in the ITSA models showed a high level of adjustment both in the global model and in the models by age group (Fig 4). In addition, once the models were adjusted, we could confirm that suicides increased significantly in Spain after the 2011 recession (p < 0.05), although this increase was now observed in both men and women.

## Discussion

Our study aimed to assess the time series pattern of age-standardised suicide rates and their association with the recent economic downturns in Spain. We used an interrupted time series analysis and adjusted this relationship by indicators of social cohesion and community values that might explain variations in suicidal trends. Based on the initial hypothesis, the analysis revealed a positive and statistically significant association between the 2011 economic downturn and suicide rates with varying patterns that were limited to specific sex and age groups. These variations have also been found at local levels in previous work [32]. Along the general association pattern for the country, both the 1992 recession and the 2011 recession were found to be associated with increasing suicides rates in adjusted models (Table 3), however, the measurement of the 2008 economic downturn still remain partially inconsistent with previous studies that found an increase in suicides during the first years of financial crisis [32–36].

The findings show that the Great Recession was positively related to the increasing suicide rates, though this statistically significant association was particularly observed after the second recession. This result corroborates that the economic crisis has possibly impacted the growing suicide rates, but exclusively during the period characterised by economic cuts after the 2011 recession and both in males and females [37]. In particular, it is interesting to observe how after the 2011 economic downturn suicide rates have increased along mid-ages groups and specially between the 45–64 years old interval, i.e. a non-young population group that might be defined by family responsibilities and possibly problem associated (e.g. loans, mortgages, children or minor on charge, family lost, etc.). However, increasing suicide rates are also common among younger and older groups, although the trends are not constant to describe a general pattern. Especially, it is striking to observe how suicide rates of the younger groups have increased after the 2011 recession.

Although it is reasonable to understand why suicide rates grew during the stronger period of economic cuts promoted by the Spanish government and the Troika, it is difficult−and perhaps risky−to provide a single explanation for the decreasing suicide rates after the 2008 recession. This reduction in suicides might be explained using different but complementary views. On the one hand, as a recent study has recently showed, suicide patterns could have decreased due to methodological changes in suicide data registry [4]. However, in the other hand, this reduction might be related with the progressive increase in population resilience and social expenditure during these first years of social and economic uncertainty [38]. For instance, the relevance of some of the social protection measures such as unemployment protection and, perhaps to a lesser extent, disability and family protection, became evident in the analysis [39]. In particular, our models showed the importance of social protection for unemployed population, a branch of expenditure that presented a negative relationship with the suicide rate in some groups (e.g., 15–24, 35–39, and 50–54 years old).

Regarding our second hypothesis, as we conjectured, social factors such as interpersonal trust, social capital or post-materialist values have been found to be associated with suicide trends during the period under analysis. Although unemployment has been frequently highlighted in specialised literature as the fundamental determinant of suicides after the 2008 economic downturn, it is difficult to confer the full explanatory potential to this economic determinant [31]. In fact, despite the clear positive effect of unemployment on the increase of suicides in the country as a whole, in Spain we can see that the regions that have the higher unemployment rates do not coincide with those where suicide is more prevalent [40], and this same idea can be applied to different age groups. Specialised literature points out to resilience in certain disadvantaged populations [38], but there may still be more factors that affect suicidal tendencies. In fact, this is a classical idea that is inherent in the Durkheim’s study [41]. In parallel to fluctuations in macroeconomic indicators (i.e. increasing unemployment, GDP declines, public and social expenditure reductions), in recent years, there have been important changes in the values of Western societies that have been traduced in the emergence of new social movements [42].

Interpersonal trust and social cohesion might have diminished in the moments of uncertainty during the 2008 Financial Crisis in Spain, while post-materialist values and subsequently individualist orientations might have increased, contrary to the results found in other works [23]. Social capital and trust have been generally positively associated with health outcomes, but in our results we can observe that social capital can also present the opposite effect, i.e. social capital was also found positively related to suicide trends among the younger and older groups. Thus, while a high social integration might present a protective effect among middle-age groups that have suffered the impact of the 2008 Financial Crisis, an excessive social cohesion can have reinforced social contagion of risk behaviors, sedentary or unhealthy lifestyles among certain communities.

Although this result can initially seem theoretically puzzling, recent studies have also described a dark side of social capital [43]. According to our results, social cohesion seems to be protective between mid-age working groups, but do not among the young and the elderly. At the same time, the positive association of suicides with increasing post-materialist values in Spain can be logic from a Durkheimian point of view. Post-materialist orientation emphasizes self-expression and quality of life over basic needs (i.e. economic and physical security), therefore suicides related with this indicator might be considered as that what has been defined as *egoistic* suicide, which is an expression of excessive individuation [41].

Finally, it is relevant to mention the generalized increase of suicide among women, which–in parallel with the male suicide decline–is particularly contributing to reduce the suicide gender gap among the younger groups, either in children, adolescent and young adults. As in other Western countries, men still die by suicide more frequently than women in Spain, however female suicide rates seem to be rising more quickly [44]. Regarding these results, a recent study has pointed out to the opioid epidemic [45] and the social media as possible causes of the youth and the female uptick in the US, but we will need specific data on these factors and a more complex model design in order to provide additional insights about the possible causes in the Spanish context. In any case, although we will require additional evidence to understand the underlining reasons of increasing suicides among the diverse social groups, our study highlights a complex reality that is difficult to address from a single perspective and also evidences the need to adopt a multidisciplinary approach in the design of policies for suicide prevention in order to implement tailored policies that protect the most vulnerable groups against suicide: females, elderly population, minors and teenagers, unemployed or suffering poor working-living conditions, people with physical or mental disabilities, and, in particular, those who are socially excluded.

### Limitations

The main limitation of our study is related with the quality suicide data. As previous studies have highlighted methodological variations in suicide data registry might derive in the underestimation of the real problem in Spain [4]. In addition, according to the ecological fallacy, we must understand that individual behavior cannot be inferred from the use of aggregate national data. In any case, apart from these limitations related with the use of complex data, our findings show highly plausible results that are consistent with previous evidence, and additionally present some advantages compared with previous research such as allowing the study of contextual changes associated with different time recession periods, diverse socio-economic determinants, and how have they impacted in national suicidal trends.

## Supporting information

S1 Table Interrupted time series analysis for suicides in Spain (full model). (DOC)
