# Supplementary material for: Structural determinants of suicide during the global financial crisis in Spain: Integrating explanations to understand a complex public health problem
Source: PLoS One. 2021 Mar 1;16(3):e0247759. doi: 10.1371/journal.pone.0247759 (PMC7920348; doi:10.1371/journal.pone.0247759)
Supplement: S1 Table — (DOC) [file pone.0247759.s001.doc]

S1 Table. Interrupted time series analysis for suicides in Spain (Full model)

| **Variables** | **Coef.** | **Std. Err.** | **t** | **P>t** | **95% CI** | |
| --- | --- | --- | --- | --- | --- | --- |
| Time | 0.001 | 0.000 | 3.290 | 0.002 | 0.000 | 0.001 |
| Sex (female=1) | 0.019 | 0.004 | 4.830 | 0.000 | 0.011 | 0.027 |
| Sex_time | 0.002 | 0.001 | 3.260 | 0.002 | 0.001 | 0.003 |
| Recession1 | -0.007 | 0.004 | -1.890 | 0.064 | -0.014 | 0.000 |
| Rec_time1 (1992) | -0.001 | 0.000 | -2.670 | 0.010 | -0.002 | 0.000 |
| Sex_time1 (1992) | -0.025 | 0.006 | -4.030 | 0.000 | -0.037 | -0.013 |
| Sex_time1_rec1 (1992) | -0.003 | 0.001 | -3.400 | 0.001 | -0.004 | -0.001 |
| Recession2 | -0.001 | 0.002 | -0.560 | 0.577 | -0.006 | 0.003 |
| Rec_time1 (2008) | -0.002 | 0.000 | -8.280 | 0.000 | -0.003 | -0.002 |
| Sex_time1 (2008) | 0.000 | 0.006 | 0.080 | 0.933 | -0.011 | 0.012 |
| Sex_time1_rec1 (2008) | 0.004 | 0.003 | 1.560 | 0.124 | -0.001 | 0.009 |
| Recession3 | 0.004 | 0.001 | 3.440 | 0.001 | 0.002 | 0.007 |
| Interv_time1 (2011) | 0.004 | 0.000 | 8.390 | 0.000 | 0.003 | 0.005 |
| Sex_time1 (2011) | -0.009 | 0.005 | -1.820 | 0.074 | -0.020 | 0.001 |
| Sex_time1_rec1 (2011) | -0.003 | 0.003 | -1.090 | 0.281 | -0.009 | 0.003 |
| Constant | 0.005 | 0.001 | 4.810 | 0.000 | 0.003 | 0.007 |
|  |  |  |  |  |  |  |
|  | Period: 1992-2008 | | Period: 2008-2011 | | Period: 2011-2017 | |
|  | Coef. | P>t | Coef. | P>t | Coef. | P>t |
| Male | -0.004 | 0.887 | -0.260 | 0.000 | 0.044 | 0.529 |
| Female | -0.006 | 0.557 | -0.108 | 0.000 | 0.063 | 0.012 |
| Difference | 0.002 | 0.941 | -0.151 | 0.005 | -0.019 | 0.798 |
